# Unrevealing the Hidden Effects of Job Insecurity: A Moderated-Mediation Model of Moral Disengagement and Moral Identity

**DOI:** 10.3389/fpsyg.2022.906896

**Published:** 2022-07-14

**Authors:** Zheng Zhang, Muhammad Waqas, Farzan Yahya, Usman A. Qadri, Joseph Marfoh

**Affiliations:** ^1^School of Management, Jiangsu University, Zhenjiang, China; ^2^School of Marxism, Changzhou College of Information Technology, Changzhou, China; ^3^Department of Business Administration and Commerce, Institute of Southern Punjab, Multan, Pakistan; ^4^Universiti Sultan Zainal Abidin, Terengganu, Malaysia; ^5^Department of Health Policy and Management, School of Management, Jiangsu University, Zhenjiang, China

**Keywords:** job insecurity, moral disengagement, social undermining, moral identity, healthcare workers

## Abstract

Moral disengagement is an intensely negative reaction that triggers unethical behavior in the workplace. By integrating the conservation of resources and moral disengagement theories, the current research examined how moral disengagement can explain the mechanism through which job insecurity results in adverse consequences. Furthermore, moral identity was theorized to moderate the hypothesized relationships. The theoretical model was tested by using time-lagged multisource data collected from 425 Chinese employees and their respective supervisors associated with the healthcare sector. The study concluded that job insecurity was positively linked with employees’ moral disengagement, which, in turn, led to coworker undermining behavior. Furthermore, moral identity moderated the relationship between job insecurity, moral disengagement, and coworker undermining such that employees high in moral identity experience less moral disengagement and are less involved in coworker undermining. Theoretical and practical implications along with future research avenues are discussed.

## Introduction

Job insecurity is defined as “the anticipation of a stressful event in such a way that the nature and continued existence of one’s job are perceived to be at risk” (Sverke and Hellgren, 2002). Given the rapid growth in globalization, the recent changes in labor markets, increased government interventions on labor market regulation, and increased pressure on organizational financial resources (Allvin et al., 2011) have all led organizations to adopt several diverse policies (Datta et al., 2010), such as downsizing and restructuring, to improve their profitability and effectiveness (Sitlington and Marshall, 2011). In turn, these policies have created an increased sense of job uncertainty among employees.

Job insecurity, or the fear of job loss, is currently considered as a psychological stressor that causes significant adverse outcomes not only among employees but also their organizations and significant others. The early research on job insecurity focused on the adverse effects on employees’ emotions and behaviors, and their physical and psychological well-being (Reisel et al., 2010; De Witte et al., 2015; Piccoli and De Witte, 2015; Wang et al., 2015). More recently, research has begun to recognize the damaging effects of job insecurity on others within organizations (Huang et al., 2016). This study focuses on one such behavior, i.e., coworker undermining behavior.

Social undermining in the workplace is defined as behavior intended to hinder, over time, a worker’s ability to establish and maintain positive interpersonal relationships, work-related success, and a favorable reputation (Duffy et al., 2002, 2006). It is not as intense as bullying or other forms of mistreatment and its consequences may not be immediately observable, but social undermining is a form of mistreatment (Aquino and Thau, 2009), that can create a toxic atmosphere within an organization (Duffy et al., 2006; Lee et al., 2016) and hence disrupts organizational operations. Earlier studies documented job insecurity as a workplace stressor with undesirable outcomes, but they also concluded that one’s reaction to any unmet expectation depends upon individual differences (Näswall et al., 2005; Paulsen et al., 2016). In line with these arguments, this study focuses on moral identity as an individual difference that moderates the relationship between job insecurity, moral disengagement, and coworker undermining behavior.

**Figure 1:**
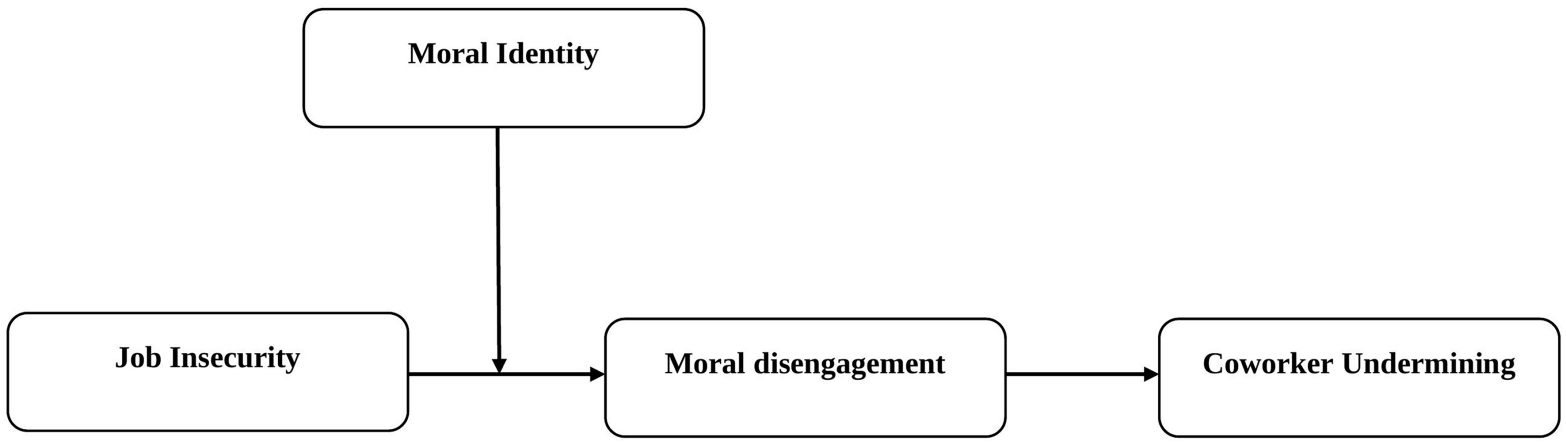
The proposal moderated-mediation model.

This study intends to fill the gaps found in prior research and offers several contributions to the existing literature. Firstly, this study highlights additional negative outcomes by concluding that job insecurity not only minimizes employees’ efforts on behalf of the organization but also increases their involvement in such activities, which then may harm the organization and others within the organization. By including social undermining in this study, we extend the research on the adverse effects of job insecurity beyond that which focus on employees’ wellbeing (De Witte et al., 2015; Chirumbolo and Hellgren, 2016), organization-related outcomes (Jamal, 1990), and the effects on families (Westman et al., 2001). Second, prior research largely focuses on personality traits (Detert et al., 2008) and emotions (Duffy et al., 2012) as antecedents of moral disengagement, whereas this study considers contextual cognition (i.e., job insecurity) as an antecedent. On this basis, we can address why employees engage in unethical behavior when they perceive job insecurity, and we focus on the perspectives of conservation of resources theory (Hobfoll, 1989) and moral disengagement theory (Bandura et al., 1996). Third, this study also adds insights into the growing literature on individual’s differences. In particular, the study concludes that a high moral identity renders an employee to less likely to engage in coworker undermining behavior. In summary, by integrating COR theory and moral disengagement theory, we address when and why job insecurity results in coworker undermining behavior. A thorough understanding of this aspect enables managers to be well positioned in managing employees’ undermining behavior. Figure 1 shows theoretical model of the study.

## Literature Review and Hypotheses Development

### Job Insecurity and Conservation of Resources

Job insecurity is considered a form of workplace stress, one that threatens employees with resource loss, which can result in undesirable outcomes (Mohr, 2000; Leka et al., 2010; Staufenbiel and König, 2010). Mental and physical well-being, job satisfaction, performance, creativity, and commitment, among others, have all been shown to be adversely affected by job insecurity (De Witte et al., 2015; Ouyang et al., 2015; Caroli and Godard, 2016). Research has also shown that job insecurity is associated with emotional reactions; e.g., anxiety, emotional exhaustion, and depression (Aaronson and Sullivan, 1998; Westman et al., 2001; Ferrie et al., 2002; Stander and Rothmann, 2010). These findings are consistent with the COR theory that argues that employees are less motivated to maintain positive attitudes when they work in environments that threaten their available resources, and they instead withdraw themselves from such environments and engage in coping processes to regain their lost resources.

Based on COR theory, we posit that job insecurity signals the threat of resource loss of employees, which then causes them to conserve, rather than invest their available resources (De Cuyper et al., 2012). We also position job insecurity as a type of injustice by an organization (Piccoli and De Witte, 2015). Individuals who experience job insecurity may perceive an imbalance between their efforts and the rewards they receive from the organization, which results in the perception of unjust treatment. Previous research has concluded that victims of resource loss or unjust treatment may act aggressively toward others (Jones, 2009; Wang et al., 2011; Lian et al., 2014; Lee et al., 2016). Based on these arguments, our framework proposes that the threat of resource loss and the perception of unjust treatment leads employees to engage in unethical behavior, i.e., social undermining behavior (Lee et al., 2016).

### Mediating Role of Moral Disengagement

According to the theory of moral disengagement (Bandura, 1986, 1991), human behaviors are molded by self-regulatory systems. Individuals exhibit ethical behavior as long as their self-regulatory system is fully functioning. Moral disengagement refers to situations where individuals fail to maintain ethical standards in regulating their behavior. The theory claims that people high in moral disengagement are more likely to cognitively deactivate their self-regulatory system, i.e., They do not feel a moral obligation to behave in a socially desired manner (Detert et al., 2008; Huang et al., 2016).

The theory of moral disengagement incorporates eight mechanisms grouped into three broad categories to explain the deactivation of self-regulation systems (Bandura, 2002). The first category refers to situations in which individuals hide the damaging effects of their behavior. This category includes the shifting of accountability, distribution of obligation, and the distortion of penalties. In this scenario, individuals do not consider themselves accountable for any wrongdoing on their part. They transfer responsibility to their leaders or others by conveying that their behavior is influenced by their leaders or pressured by significant others. For example, consider a group of salespersons who did not achieve their sales targets. In this situation, individuals can free themselves from responsibility by assuming that the entire group is responsible for this failure, not any single individual. The second category refers to situations in which individuals come up with moral reasons for their decadent actions. This category includes moral reasoning, euphemistic labeling, and comparative advantage. For example, an individual caught stealing food items from a store may proclaim himself or herself to be innocent because he or she had not eaten food for several days, and stealing was the last option. Such an explanation provides the person with a moral reason for criminal activity. The third category refers to situations in which individuals reduce their moral identification with the target, which purportedly allows them to behave unethically toward others by assuming that the target individual deserves the mistreatment. For instance, the police may falsely arrest an individual for recent criminal activity on the simple assumption that the person has a past criminal record. All three mechanisms of moral disengagement are closely interlinked and operate in unison (Christian and Ellis, 2014).

Previous work tested moral disengagement in military and political settings (Aquino et al., 2007; Jackson and Gaertner, 2010; Leidner et al., 2010). Attention has also been given to the effects of moral disengagement on offspring and teenagers (Hymel et al., 2005; Gini et al., 2014). The current study examines the role of moral disengagement in the workplace by exploring its impact on employees’ behaviors. Based on prior research which found that moral disengagement provides a basis for unethical behavior (Barsky, 2011; Claybourn, 2011; Moore et al., 2012), we propose that job-insecure employees attribute negative experiences, at least in part, to actions taken by others. These employees perceive the actions of management and their co-workers have created a job insecure-environment for them and this enables them, from which the said employees associate moral reasons for their unethical behavior. As we reasoned, job insecurity creates the threat of resource loss, which then causes the victims to perceive being treated unjustly. The individuals are also compelled to regulate their actions, thus leaving them with fewer resources and deepened feelings of unjust treatment. However, job-insecure employees need moral reasons to justify their potential undermining behavior. On this basis, we propose that they avoid self-blame through moral disengagement. Job-insecure employees consider undermining behavior as justifiable for taking revenge against all those who contributed to their job insecurity. Thus, moral disengagement frees employees from their moral obligations. Consequently, we propose that moral disengagement determines the degree to which job insecurity leads to coworker undermining.

*Hypothesis 1:* Moral disengagement mediates the positive relationship between job insecurity and employees’ coworker undermining behavior.

### Moderating Role of Moral Identity

Moral identity is described as “the degree to which being a moral person is important to an individual’s identity” (Hardy and Carlo, 2011). It is also considered as a motivation for exhibiting prosocial interaction (Hardy and Carlo, 2011; Wang et al., 2017), which results in the morally desired behavior (Reed and Aquino, 2003; Winterich et al., 2013; Hertz and Krettenauer, 2016).

Aquino and Reed (2002) found that moral identity enhances an individual’s self-regulation and fosters moral actions. Based on this argument, we believe that moral identity has the potential to reduce the undesirable behavioral outcomes associated perception of unjust treatment (i.e., moral identity renders the outcomes less effective). Two reasons are considered for proposing a moral identity as a potential moderator in our study. First, individuals for whom moral beliefs are important to consider moral obligations, and they show concern for others’ needs and values. Consequently, such individuals attempt to act in socially desired ways so that people around them will not be offended by their behavior (Winterich et al., 2009; Wang et al., 2017). Second, individuals with high moral values incorporate a wider array of ethical actions and consider the suffering that their behavior may cause, and thus, they are encouraged to act more ethically. Several studies have already confirmed the role of moral identity in restraining unethical behavior (Eisenberg^*^, 2004; West et al., 2004) and shown that individuals who are high in moral identity are less likely to show anger and aggression (Aquino et al., 2007). With these arguments, we propose that employees with high moral identity are less likely to experience moral disengagement, and ultimately less involve in coworker undermining behavior.

*Hypothesis 2a:* Moral identity moderates the direct positive relationship between job insecurity and moral disengagement, such that the relationship will be weaker for those employees who are high in moral identity.

*Hypothesis 2b:* Moral identity moderates the indirect positive relationship between job insecurity and coworker undermining through moral disengagement, such that the mediated relationship will be weaker for those employees who are high in moral identity.

## Methodology

### Sample and Procedure

By using a convenience sampling technique, the data were collected from employees associated with the healthcare sector in china. Assistance was received from the organization’s human resource department to announce the study along with a letter that assured confidentiality and voluntary participation. In particular, employees were assured that their supervisors would not know their responses to the survey.

The data were collected in two phases. In the first phase, employees completed a survey on demographics and perceived levels of job insecurity. After 1 month, we conducted another survey that asked about employees’ moral identity and moral disengagement. At the same time, 26 supervisors were contacted to report their subordinates’ coworker undermining behavior. All the surveys were distributed during work hours. The participants completed the surveys while at work, and they were asked to submit their completed surveys directly to the authors. This procedure reassured the participants that their survey responses would remain undisclosed to others. The scales we used were developed in English, and thus, we translated them into Chinese; for back-translation, we enlisted the assistance of two Chinese bilingual academicians to confirm the quality and accuracy of the translation (Brislin, 1980). Since our research model had four variables and a total of 37 items, the minimum size of the sample required for our study was 148 (37 × 4 = 148). The size of the sample used in our study (i.e., *N* = 425) is larger than the required sample size and which is adequate enough for analysis and give more reliable results with greater precision and power, as suggested by Benner and Waldfogel (2008).

### Measures

#### Job Insecurity

A four-item scale developed by De Witte (2000) was used to measure employee perceptions of job insecurity. Sample items include “I am sure that I will be able to keep my job” (reverse coded) and “I feel uncertain about the future of my job.” Responses were rated on a 5-point Likert scale (1 = strongly disagree, 5 = strongly agree).

#### Moral Disengagement

Moral disengagement was measured using McFerran et al. (2010) scale previously used by Duffy et al. (2012). The scale includes 15 items, including “People mistreated at work have usually done something to deserve it,” and “Making fun of your coworkers does not hurt them.” The responses were anchored on a 5-point Likert scale (1 = strongly disagree, 5 = strongly agree).

#### Moral Identity

The moral identity scale developed by Aquino and Reed (2002) was used to measure the respondent’s moral identity. Participants were given 13 adjectives and asked to indicate how much they desired to have each of these characteristics. Adjectives included: care for others, being friendly, passionate, generous, piousness and kindness. The construct is based on thirteen items; the sample item includes questions like “Being someone who has these characteristics is an important part of who I am,” and “I am actively involved in activities that communicate to others that I have these characteristics.” Respondents were asked to record their responses on a 5-point Likert scale (1 = strongly disagree, 5 = strongly agree). Higher the score higher would be the moral identity of respondents.

#### Coworker Undermining

Coworker undermining was measured by a 5-item scale (Kammeyer-Mueller et al., 2013). The unit supervisors were asked to indicate the extent to which their subordinates are involved in social undermining behavior at the individual level. Sample items include “Criticizes his/her colleagues,” and “Acts unpleasantly or angrily toward others.” The responses were anchored on a 5-point Likert scale (1 = to no extent, 5 = to great extent), where higher scores indicate stronger employee involvement in undermining behavior.

#### Control Measures

Consistent with other studies (Sora et al., 2010; Selenko and Batinic, 2013; Lam et al., 2015), we controlled for demographic variables in an attempt to measure theorized hypotheses accurately. According to the gender-role socialization theory (Eagly, 2013) women tend to portray themselves as emotionally expressive and interpersonally connected, while men represent themselves as independent and self-reliant. Because gender differences may explain the difference between male and female responses to job insecurity, we controlled for employee gender. Additionally, we controlled for employee organizational tenure (Lam et al., 2015) because prior studies suggested that employees with longer tenures have devoted a major part of their career to the organization, leaving them more emotionally attached and committed to the organization and therefore are more likely to react strongly to job insecure conditions (Kuhnert and Vance, 1992). Additionally, Cheng and Chan (2008) concluded that older employees are often more affected by negative outcomes of job insecurity as they have more family responsibilities, lower occupational mobility and are more dependent on their current job, so job insecurity has more adverse consequences for them. Consequently, we controlled for age as well. Regarding education, individuals who are highly educated have greater expectations from their employers and if their expectations are not met they are more dissatisfied compared to less educated employees in similar circumstances (Coyle-Shapiro and Kessler, 2000). On the other hand, research also suggests that highly educated employees consider themselves superior with more human capital and they perceive greater job opportunities if they get laid off (e.g., De Witte and Näswall, 2003; Hellgren and Sverke, 2003). With higher employability, highly educated employees are less susceptible to the effects of job insecurity. Since education has contradictory effects, we followed previous research (Berntson et al., 2010; Otto et al., 2011) and controlled for its effect in our analysis. Finally, for contract type, research suggested that different contracts hold different expectations from the employer. Moreover, different contracts involve different levels of job security, so their discrete impact should be assessed considering the appropriate set of expectations as a reference (Cuyper and Witte, 2006). In summary, following prior studies (Richter et al., 2014; Wang et al., 2014), we controlled for employee gender, age, education, tenure, and contract type.

## Analysis of Results

For this study, the statistical tool is divided into two categories, descriptive statistics, and inferential statistics.

### Descriptive Statistics

#### Reliability, Correlations, Discriminant, and Convergent Validity of the Constructs

In the study sample, the majority of respondents 255 (60%) were male, 170 (40%) were females, 275 (65%) were from the age limit 31–40 years, 290 (68%) had a bachelor’s degree, and the majority of the respondents 285 (67%) spent 5–10 years in an organization. Before assessing the study’s hypotheses, we first test the internal consistency of all the study constructs. To determine the internal consistency of the instrument items, we employed coefficient alpha (*α*). In Table 1, the *α* value of 0.70 of each study constructs above 0.70 which is considered acceptable, as suggested. In addition, confirmatory factor analysis (CFA) *via* AMOS was used to examine the measurement’s convergent and discriminant validity.

**Table 1:** Mean, standard deviation, correlations, reliability, and validity of the variables.

Variables	1	2	3	4	5	6	7	8	Mean	SD	Kurtosis	Skewness	*α*	AVE	MSV
1. Job insecurity	**(0.768)**	0.536^**^	0.351^**^	0.284^**^	−0.007	−0.023	0.022	0.098^*^	3.218	1.257	−1.246	−0.066	0.804	0.590	0.438
2. Moral disengagement		**(0.780)**	0.519^**^	0.394^**^	0.012	−0.023	0.014	−0.009	3.024	1.232	−1.254	−0.044	0.822	0.609	0.438
3. Coworker undermining			**(0.837)**	0.516^**^	0.009	−0.057	−0.009	−0.026	2.939	1.282	−1.289	−0.140	0.940	0.701	0.361
4. Moral identity				**(0.742)**	0.016	−0.012	−0.019	−0.035	3.169	1.161	−1.080	−0.232	0.726	0.551	0.332
5. Qualification					–	0.013	0.059	−0.001	1.889	0.553	0.142	−0.047	–	–	–
6. Gender						–	−0.009	−0.083	1.372	0.484	−1.725	0.533	–	–	–
7. Organization tenure							–	−0.017	2.489	0.727	−0.253	0.314	–	–	–
8. Age								–	2.551	0.878	−0. 715	0.044	–	–	–

*n = 425; SD, Standard Deviation; MSV, Maximum Shared Variance; AVE, Average Variance Extracted; α, Cronbach’s alpha*

Statistically Significant at: ^**^p < 0.001, ^*^p < 0.05; the Data on the diagonal (in bold) is the square root of AVE of the constructs

1 *Gender: 1 = Male, 2 = Female;*

2 *Age: 1 = 18–30 years old, 2 = 31–40 years old, 3 = 41–50 years old, 4 = over 50 years old;*

3 *Qualification: 1 = Understand Graduate, 2 = Graduate, 3 = Post Graduate;*

4 *Organization Tenure: 1 = less than 1 year, 2 = 1–5 years, 3 = 6–10 years, 4 = more than 10 years*.

Table 1 shows the mean value for all constructs ranged from 1.372 to 3.218, the SD 0.484 to 1.257, the values for kurtosis is between – 3 to +3, and the skewness is between −2 to +2 for the all variables, which are under the acceptable range as suggested by Hair et al. (2010).

For better results, factor loadings/regression weights should be 0.50 or higher in the measurement model, as suggested by Hair et al. (2010). Therefore, research items with regression weights <0.50 were discarded and further not included in the analysis. For instance, items like, “Talking about people behind their backs is just part of the game” are dropped from the analysis due to regression weights less than the threshold limit. Consequently, 37 out of 27 items from the study instrument were extracted using CFA, and 10 items were rejected due to factor loadings <0.50, as recommended by Hair et al. (2010). Besides, the study measurement model provided the numerous model-fit indexes: CMIN/DF = 1.876 < 3.0, “Goodness-of-Fit Index” (GFI) = 0.955 > 0.90, “Root Mean Square Error of Approximation” (RMSEA) = 0.045 < 0.60, “Adjusted Goodness-of-Fit Index” (AGFI) = 0.930 > 0.90, “Comparative Fit Index” (CFI) = 0.983 > 0.90, and “Non-normed fit index” (NNFI) = 0.964 > 0.90. As shown in Table 1, MSV values < AVE for each construct (i.e., discriminant validity), and AVE of each construct is >0.50 (i.e., convergent validity), which confirms that the study measurement model has no discriminant and convergent validity issues, as recommended by Byrne and Van de Vijver (2010).

Table 1 shows that there were the significant and positive correlations between Job insecurity & moral disengagement at (*r* = 0.536, *p* < 0.001), Job insecurity & coworker undermining at (*r* = 0.351, *p* < 0.001), Job insecurity & moral identity at (*r* = 0.284, *p* < 0.001), moral disengagement & coworker undermining at (*r* = 0.516, *p* < 0.001), moral disengagement & moral identity at (*r* = 0.394, *p* < 0.001), and coworker undermining & moral identity at (*r* = 0.516, *p* < 0.001). Overall, in the correlation matrix there were not unexpected results.

### Inferential Statistics

Despite the fact that research participants worked in various institutional contexts, subordinates in the same office report to the same supervisor. As a result, the findings of OLS regression might result in erroneous test statistics or biased standard error estimates. This study assessed intraclass coefficient 1 (ICC1, variance between supervisors) and intraclass coefficient 2 (ICC2, supervisors’ means’ stability) to determine the appropriate degree of analysis before testing our empirical model. The intra-class coefficients (ICC1s) for Job Insecurity, Moral Disengagement, Moral Identity, and Coworker Undermining were 0.19, 0.18, 0.14, 0.15 and 0.22 while ICC2s were 0.20, 0.29, 0.09, 0.21 and 0.25, respectively. All these coefficient values are below Cicchetti’s (1994) acceptable range of 0.by 7 as suggested Cicchetti’s (1994), which allows us to use the multilevel method.

In addition, this study calculated a corrected F statistic to further corroborate to the study findings, and all of the values are significant, with no value decreasing by 0.10. Moreover, this study analyze all of the constructs in the study model at the individual level, following Kenny's (1995) recommendation that individual-level analysis can be estimated when intraclass coefficients are less than 0.3.

To test the study hypotheses, we used the SPSS PROCESS macro developed by Preacher and Hayes (2004) to conduct data analysis, as shown in (e.g., Eissa and Lester, 2017; Hongbo et al., 2020, 2021). Preacher and Hayes (2004) described a set of investigations that we used to develop formal mediation, moderation, and moderated-mediation hypotheses. First, to determine the significance of the mediation, we used the Hayes “PROCESS macro” (Hayes and Rockwood, 2017) to construct a bias-corrected CI by bootstrapping (with 5,000 bootstrap subsamples). The formal mediation hypothesis was also tested using the Sobel test (normal theory method) by the Hayes PROCESS macro. Second, using the Hayes PROCESS macro, we were able to assess the moderated mediation model’s index as well as the conditional indirect effects of Job insecurity on coworker undermining *via* Moral disengagement at different Moral identity levels (+1SD, Mean, and −1SD). We also used Edwards and Lambert (2007)’s technique, along with Hayes “Process Macro,” to run a simple slop test and generate a graph to assess the moderated-mediation model (two-way interaction). To test H1, we first employed the SPSS “PROCESS macro” Model 4 in SPSS (i.e., mediation hypothesis). We then used “PROCESS macro” Model 7 *via* SPSS to test the proposed moderated-mediation model (i.e., H2a & H2b).

#### Mediation Tests

Table 2 presents the findings of the formal mediation test. Job insecurity is positively related with moral disengagement (*β* = 0.531, SE = 0.041, *t* = 13.108, LLCI = 0.451, ULCI = 0.612) and coworker undermining (*β* = 0.109, SE = 0.050, *t* = 2.150, LLCI = 0.009, ULCI = 0.208). moral disengagement is positively related with coworker undermining (*β* = 0.480, SE = 0.051, *t* = 9.356, LLCI = 0.379, ULCI = 0.581) as well. Following the recent studies (e.g., Tariq and Ding, 2018; Popelnukha et al., 2021), we calculate bias-corrected bootstrapped CIs (using 5,000 bootstrap subsamples) for indirect effects of job insecurity on coworker undermining through moral disengagement. Table 2 illustrates the statistically significant positive direct effects of job insecurity on coworker undermining (*β* = 0.109, SE = 0.051, LLCI = 0.009, ULCI = 0.208), indirect effects of job insecurity on coworker undermining *via* moral disengagement (*β* = 0.255, SE = 0.036, LLCI = 0.187, ULCI = 0.328), and the total effects of job insecurity on coworker undermining (*β* = 0.363, SE = 0.047, LLCI = 0.272, ULCI = 0.455) provide support for **H1**; that is, the positive association among job insecurity and coworker undermining is mediated by moral disengagement.

**Table 2:** Results of mediation analysis.

Antecedents	Moral disengagement						Coworker undermining					
	Std. **β**	Std. error	*t*-value	LLCI	ULCI	*R* ^2^	Std. *β*	Std. error	*t*-value	LLCI	ULCI	*R* ^2^
						0.292^***^						0.281^***^
Constant	1.532	0.346	4.433^***^	0.853	2.211		1.503	0.371	4.049^**^	0.773	2.233	
Job insecurity	0.531	0.041	13.108^***^	0.451	0.612		0.109	0.050	2.150^*^	0.009	0.208	
Moral disengagement	–	–	–	–	–		0.480	0.051	9.356^***^	0.379	0.581	
*Control variables* Gender	−0.0412	0.105	−0.392	−0.248	0.165		−0.124	0.110	−1.121	−0.341	0.093	
Age	−0.089	0.058	−1.531	−0.203	0.025		−0.053	0.061	−0.866	−0.173	0.067	
Qualification	0.035	0.092	0.388	−0.145	0.216		0.014	0.096	0.1440	−0.176	0.203	
Organization Tenure	−0.000	0.070	−0.003	−0.138	0.137		−0.034	0.073	−0.468	−0.178	0.110	
Predicator			Std. β		Std. Error			LLCI			ULCI	
Direct effects												
Job insecurity on coworker undermining			0.109		0.051			0.009			0.208	
*Indirect effect*												
Job insecurity on coworker undermining *via* Moral disengagement			0.255		0.036			0.187			0.328	
*Total effect*												
**Job insecurity on coworker undermining**			0.363		0.047			0.272			0.455	
**Normal theory tests for indirect effect**			**Std. β**		**Std. error**						**Z**	
Job insecurity on coworker undermining *via* Moral disengagement			0.254		0.035						7.255^***^	

Results of total, direct, indirect, and normal theory effects. N = 425; statistically Significant at: ^***^p < 0.0001, ^**^p < 0.001, and ^*^p < 0.05; LLCI, Lower-level confidence intervals at 95%; ULCI, Upper-level confidence intervals at 95%.

#### First Stage Moderation, and Moderated-Mediation Tests

**Figure 2:**
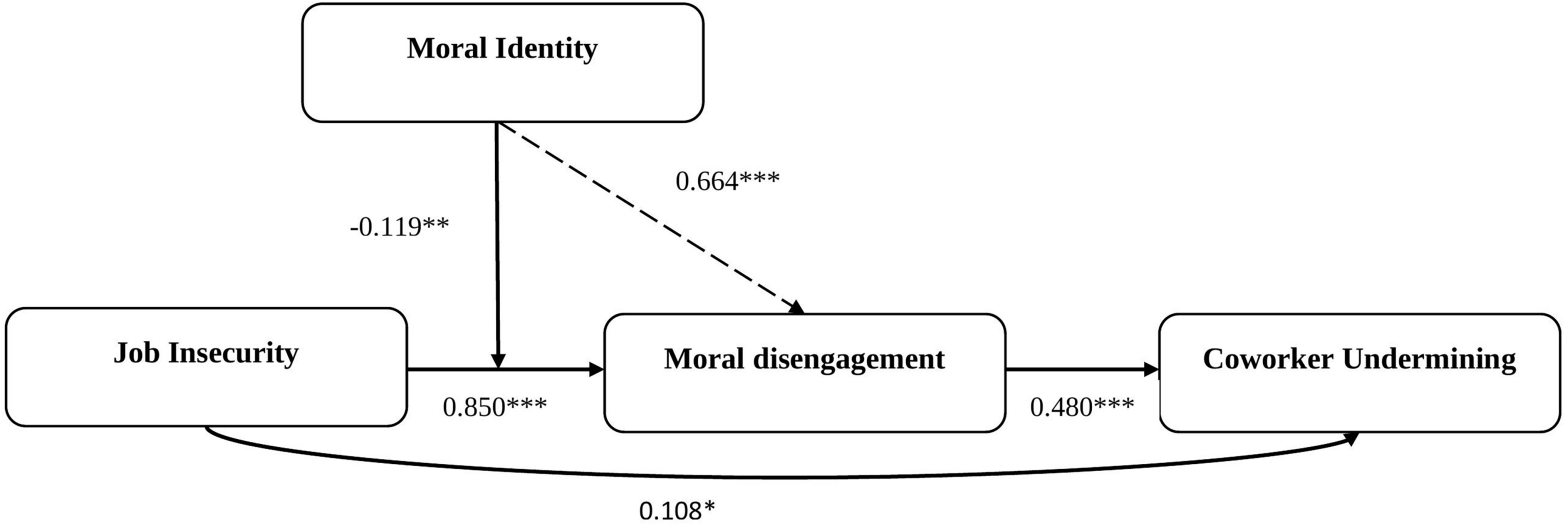
Results of moderated-mediation model. The Solid lines show the Hypothesized relationship; Statistically significant at: ^***^p < 0.0001, ^**^p < 0.001, ^*^p < 0.05.

Figure 2 and Table 3 list the results of our formal moderated-mediation model (two-way interaction). We found that job insecurity is positively related with moral disengagement (*β* = 0.850, SE = 0.128, *t* = 6.665, LLCI = 0.599, ULCI = 1.101) and coworker undermining (*β* = 0.109, SE = 0.051, *t* = 2.150, LLCI = 0.009, ULCI = 0.208). Moral disengagement is also positively related with coworker undermining (*β* = 0.480, SE = 0.051, *t* = 9.356, LLCI = 0.379, ULCI = 0.581). The interaction term of Job insecurity and Moral identity is significant and negative (*β* = −0.119, SE = 0.037, *t* = −3.246, LLCI = −0.191, ULCI = −0.047), as shown in Table 3. Therefore, H2a is supported. In addition, the results show that control variables (*viz.*, gender, age, qualification, and organization tenure) has no significant effect on key outcomes (see Tables 1–3).

**Table 3:** Results of the moderated-mediation model analysis.

Antecedents	Moral disengagement						Coworker undermining					
	Std. *β*	Std. error	*t*-value	LLCI	ULCI	*R* ^2^	Std. *β*	Std. error	*t*-value	LLCI	ULCI	*R* ^2^
						0.370^***^						0.530^***^
Constant	−0.414	0.513	−0.807	−1.423	0.595		1.503	0.371	4.049^**^	0.773	2.233	
Job Insecurity	0.850	0.128	6.665^***^	0.599	1.101		0.109	0.051	2.150^*^	0.009	0.208	
Moral Disengagement	–	–	–	–	–		0.480	0.051	9.356^***^	0.379	0.581	
Moral Identity	0.664	0.127	5.221^***^	0.414	0.914		–	–	–	–	–	
Job insecurity × Moral Identity	−0.119	0.037	−3.246^**^	−0.191	−0.047		–	–	–	–	–	
*Control Variables* Gender	−0.045	0.100	−0.450	−0.240	0.151		−0.124	0.110	−1.121	−0.341	0.093	
Age	−0.075	0.055	−1.358	−0.184	0.034		−0.053	0.061	−0.866	−0.173	0.067	
Qualification	0.032	0.087	−0.371	−0.139	0.203		0.014	0.096	0.144	−0.176	0.203	
Organization Tenure	0.020	0.066	0.299	−0.110	0.150		−0.034	0.073	−0.468	−0.178	0.110	

n = 425; Statistically significant at: ^***^p < 0.0001, ^**^p < 0.001, and ^*^p < 0.05; LLCI, Lower-Level Confidence Intervals at 95%; ULCI, Upper-Level Confidence Intervals at 95%.

**Figure 3:**
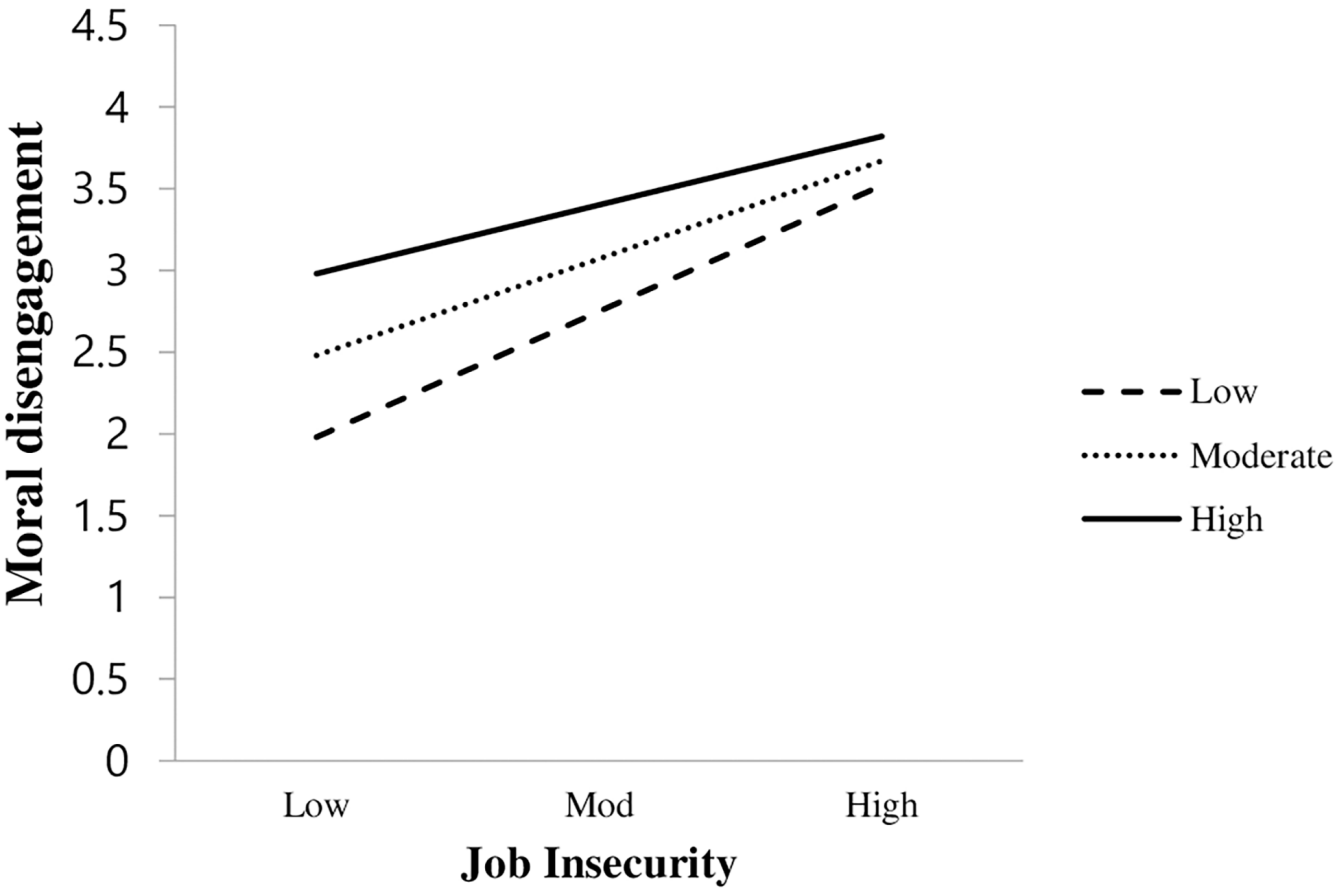
Interactive effect of Job Insecurity and Moral identity on Moral disengagement.

Besides, we followed the work of Hongbo et al. (2021); and Shillamkwese et al., (2020) to plot the first-stage moderating effect of Moral identity on the relationship between Job insecurity and Moral disengagement. By doing so, we found that the association between Job insecurity and Moral disengagement is stronger when Moral identity is low (*β* = 0.601, *t* = 9.740, *p* < 0.0001) and weaker when Moral identity is high (*β* = 0.335, *t* = 6.156, *p* < 0.0001). Concurrently, we again found support for **H2a**; that is, the positive association between Job insecurity and Moral disengagement is moderated by moral identity, such that this link is weaker when Moral identity is high. Also, to further support **H2a**, we plot the interaction term, i.e., Job insecurity × Moral identity, and provide the graphical presentation of the moderating effect of positive moral identity. Figure 3 shows that positive moral identity moderates the positive association between job insecurity and moral disengagement; such that the positive link would be weaker when positive moral identity is high.

To test **H2b**, we investigated the conditional indirect effects of job insecurity on coworker undermining *via* moral disengagement at different values of moral identity (+1SD, Mean, and -1SD). Table 4 indicates that the indirect effect of job insecurity on coworker undermining through disengagement is weak when moral identity is high (*β* = 0.161, SE = 0.035, LLCI = 0.094, ULCI = 0.233). This effect is strong when moral identity is low (*β* = 0.294, SE = 0.039, LLCI = 0.218, ULCI = 0.373). Moreover, the index of the moderated mediation is significant and negative (index = −0.057, SE = 0.018, LLCI = −0.093, ULCI = −0.023). Thus, our moderated mediation association (i.e., **H2b**) is supported; that is, the indirect positive link between job insecurity and coworker undermining *via* moral disengagement is moderated by moral identity, such that the mediated relationship is weaker when moral identity is high.

**Table 4:** Results of conditional indirect effects and total conditional effects of job insecurity on coworker undermining at values of moral identity.

Predictor	Mediator	Moderator	Std. **β**	Std. error	LLCI	ULCI
**Conditional indirect effects**						
Job insecurity on Coworker undermining	Moral disengagement	Moral Identity at –1SD	0.612	0.062	0.490	0.735
Job insecurity on Coworker undermining	Moral disengagement	Moral identity at Mean	0.474	0.040	0.395	0.554
Job insecurity on Coworker undermining	Moral disengagement	Moral identity at +1SD	0.336	0.055	0.229	0.444
**Total conditional indirect effects**						
Job insecurity on Coworker undermining	Moral disengagement	Moral identity at –1SD	0.294	0.039	0.218	0.373
Job insecurity on Coworker undermining	Moral disengagement	Moral identity at Mean	0.228	0.031	0.167	0.291
Job insecurity on Coworker undermining	Moral disengagement	Moral identity at +1SD	0.161	0.035	0.094	0.233
**Index of moderated mediation**			**Index**	**Std. error**	**LLCI**	**ULCI**
	Moral disengagement		−0.057	0.018	−0.093	−0.023

*n* = 425; LLCI, Lower-level confidence intervals at 95%; ULCI, Upper-level confidence intervals at 95%.

## Discussion

A report based on Amazon’s workplace practices highlights a highly competitive environment where employees purposely harmed their colleagues to achieve high yearly rankings (Lee et al., 2016), which support the notion that social undermining is common in highly competitive workplace settings (Lee et al., 2016). Prior research noted that relative to other forms of mistreatment such as bullying, harassment, and physical aggression, undermining behaviors are subtle low-intense forms of aggression with consequences that are often not immediately obvious (Duffy et al., 2012; Lee et al., 2016), which suggests that it is relatively easy for individuals not to be punished (Lee et al., 2016). Incidentally, victims of coworker undermining ultimately become the perpetrators of mistreatment (Aquino and Thau, 2009), which spreads toxicity throughout the organization over time (Kammeyer-Mueller et al., 2013; Lee et al., 2016). Given the severity of its negative effects, such as health issues, turnover, and reduced productivity, at the individual levels (Duffy et al., 2006; Kammeyer-Mueller et al., 2013; Lee et al., 2016), an in-depth examination is needed to answer why employees exhibit undermining behavior at work and subsequently identify the factors that can affect the strength of this relationship. To answer this argument, this study considers job insecurity as a possible antecedent of social undermining at the workplace.

A survey conducted by the American Psychological Association in 2014 found that job insecurity is one of the top five sources of job stress, e.g., 38% of the total respondents reported that job insecurity had significantly increased their work stress (Jiang, 2017). Another research also found that job insecurity affects workers worldwide (Anderson and Pontusson, 2007; König et al., 2011). Our findings highlight the importance of understanding the negative effects of job insecurity and its diffusion from one employee to others (Westman et al., 2001). Ample research has been directed toward identifying the adverse consequences of job insecurity at both the individual and organizational levels (Silla et al., 2016; Chih et al., 2017; Jiang and Probst, 2017); however, the mechanism that explains how job insecurity affects others within the organization, i.e., coworker, is an important aspect in the job insecurity literature which has remained largely unexplored. To address this research gap, our study extends the focus of prior research and examines a moral mechanism to explicate how job insecurity affects individuals’ moral consideration of others and their subsequent undermining behavior.

This study tested a moderated mediation conceptual framework in a sample of employees associated with the healthcare sector. Prior studies have also examined the adverse effects of job insecurity (De Witte et al., 2015; Sender et al., 2016; Jiang, 2017). By contrast, we tested a new mechanism to explain the diffusion of the negative effects of job insecurity from one employee to another. Our findings depict the implications of job insecurity that are even more severe than those highlighted by previous research. The results are in line with our proposed theoretical relationships, in which job insecurity (i.e., a threat to resource loss Sender et al., 2016) and the perception of injustice treatment (Piccoli and De Witte, 2015) causes undermining behavior among co-workers through the mediating effect of moral disengagement (Lee et al., 2016). This study also considered the individual difference that shapes the employees’ response to job insecurity. By considering moral identity, this study lends support to prior research showing that employees exposed to job insecurity do not respond the same way (Roskies et al., 1993; Mak and Mueller, 2000). Our findings demonstrate the prevalence of boundary conditions that surround the use of moral disengagement in response to job insecurity and the use of co-workers undermining behavior as a response mechanism. In particular, our findings show that the adverse effects of job insecurity are comparatively weak among employees who are high in moral identity.

## Theoretical Contribution

Our study adds new insights into the existing literature. First, this study extends the literature on the adverse consequences of job insecurity by examining its effects on co-workers; by contrast, prior studies focused on the employee- and organization-related outcomes (Chirumbolo and Hellgren, 2016; Chih et al., 2017). We conclude that job insecurity boosts employees’ engagement in coworker undermining behavior, and ignored but an important aspect in the job insecurity literature. By integrating COR theory and moral disengagement theory, this study proposes that employees assume that their job insecurity has stemmed from organizational policies and co-workers’ actions (i.e., their co-workers are equally responsible for their job uncertainty). This theoretical explanation provides a basis to expect the onset of undermining behavior in the workplace as a possible outcome of job insecurity. Furthermore, this study provides a novel explanation (i.e., moral disengagement) as to how job insecurity results in coworker undermining behavior. While previous studies have shown the individual’s personality and emotions as antecedents of moral disengagement (Detert et al., 2008; Duffy et al., 2012), we considered the organization’s contextual environment as the antecedent. The use of COR and moral disengagement theories demonstrates that job insecurity threatens employees’ resource loss which results in moral disengagement in the workplace.

We assume that job insecurity has detrimental effects; however, not all employees respond with the same intensity. Individual differences affect the strength of direct/indirect relationships between job insecurity and employee behavior. In particular, we examined in this study the moderating effect of moral identity. The findings revealed that moral identity moderates the relationship between job insecurity, moral disengagement, and coworker undermining. Overall, by examining the moderating effect of moral identity, we found that individuals who are high in moral identity are less likely to experience moral disengagement which ultimately reduces coworker undermining.

## Practical Implications

This study, which examined the effect of job insecurity on co-workers, i.e., coworker undermining behavior, was able to highlight additional organizational hazards. Organizations with employees who perceive threats of job loss not only face risks of reduced performance and turnover of qualified employees, but they also face problems of increasingly destructive behavior in the workplace. Such employees’ behavior is not only damaging to the organization but also threatening to co-workers. We suggest that organizations facing undermining issues should first identify its cause. Here, we find that job insecurity is one of the possible antecedents of co-workers’ undermining behavior. The implementation of the practices such as involvement of employees in decision-making processes, clear communication of challenges faced by organizations, enhanced leader-member exchange, and peer support, can limit the adverse effects of job insecurity and subsequently enhance the working environment (Bussing, 1999; Probst, 2005; Vander Elst et al., 2010; Cheng et al., 2012; Schreurs et al., 2012).

Our findings demonstrated that employees with high moral identity are less likely to engage in coworker undermining behavior, which implies that interventions should be designed to enhance employees’ moral values. This action can be done in two ways. One way is to develop and maintain a culture of high moral values, and this starts with the hiring practices. During the hiring process, companies should evaluate a candidate’s character and moral fit with the company’s work environment. For instance, during the interviews, they should ask questions that can evaluate the degree to which a candidate shares the company’s values. As mentioned above, realistic previews about working in a company with high moral values may encourage employees with different sets of values to opt-out of the organization. Furthermore, open communication within the organization, ethical leadership, and a reward system that reinforces ethical behaviors are also crucial factors in building a high-moral culture. The second way is to focus on minimizing unethical behaviors. While less desirable, a strong disciplinary system that controls unethical behaviors, even those that are less obvious and less intense, such as social undermining, creates a sense of fear which can reduce perceived freedom associated with doing something wrong.

## Limitations and Future Research Directions

The contributions of this study must be viewed in light of its limitations. The first limitation deals with the sampling technique. First, although the study is based on multi-source data, the potential problem of common method variance remains in terms of job insecurity- a moral disengagement relationship. Future researchers may opt for other data collection techniques (e.g., third-party observation) to minimize the potential CMV issue.

Second, the results revealed that the positive relationship between job insecurity and coworker undermining was mediated by moral disengagement. We believe that moral disengagement may not be the only mechanism that explains how job insecurity results in social undermining behavior. Our findings open new research avenues for other explanatory mechanisms (e.g., negative affect, emotional exhaustion, ego depletion, etc.) that can better explain the relationship between job insecurity and undermining behavior. Furthermore, there exists the possibility of parallel mediating factors that may interact with moral disengagement in transmitting the effects of job insecurity.

Third, we considered only one individual difference, i.e., moral identity, in this study. It is plausible that other personality traits or individual differences can also moderate the correlation between job insecurity, moral disengagement, and undermining behavior. Similarly, other contextual factors may also motivate employees to behave ethically. For instance, an ethical leadership style can reduce employee stress levels, which eventually can decrease unethical behavior (Mayer et al., 2010). Fourth, our study did not control other sources of stress which may have intensified employees’ stress levels, e.g., family–work conflict (Jeffrey Hill et al., 2008), abusive supervision (Bamberger and Bacharach, 2006), among others. We encourage researchers to reexamine our theoretical model control parameters for other stress sources.

Lastly, our proposed conceptual framework was tested in China, and thus, our results cannot be generalized for other cultures. According to Hofstede (1980), individuals in collectivist cultures are more concerned with job security and good working conditions, whereas their counterparts in individualist cultures prefer autonomy and task variety (Probst and Lawler, 2006). Based on cultural differences, Probst and Lawler (2006) suggested that collectivist employees can experience more negative attitudes compared with others in individualist cultures. Given that we cannot generalize our findings across different cultures, future researchers are encouraged to check if the findings remain the same in other cultures to overcome the generalizability issue.

## Conclusion

The increased tendencies of contractual employment heightened negative effects of globalization, and increased economic pressures faced by organizations have rendered job insecurity an important issue among present-day organizations. Our findings highlight more distinct effects of job insecurity than what has been previously recognized. By incorporating the mediating effect of moral disengagement, our study found that job insecurity encourages employees to engage in social undermining behavior in the workplace. Furthermore, our study extends the role of individual differences in the research on job insecurity. The one individual difference (moral identity) highlighted in our conceptual framework can be regarded as the key factor affecting the strength of the relationship between job insecurity, moral disengagement, and coworker undermining. Our findings can assist managers in designing policies that will reduce the perception and destructive consequences of job insecurity.

## Data Availability Statement

The raw data supporting the conclusions of this article will be made available by the authors, without undue reservation.

## Ethics Statement

This study was carried out following the recommendations and approval of the Ethics Committee at the Jiangsu University, China.

## Author Contributions

All the listed authors made a substantial, direct, and intellectual contribution to the work and approved it for publication. All authors contributed to the article and approved the submitted version.

## Funding

The research is supported by: General items of the Ideological and Political Theory curriculum teachers’ research projects in Colleges and universities of the Ministry of Education, 2020: Research on college students’ sense of gain from Ideological and Political theory curriculum in Colleges and Universities (No. 20JDSZK022).

## Conflict of Interest

The authors declare that the research was conducted in the absence of any commercial or financial relationships that could be construed as a potential conflict of interest.

## Publisher’s Note

All claims expressed in this article are solely those of the authors and do not necessarily represent those of their affiliated organizations, or those of the publisher, the editors and the reviewers. Any product that may be evaluated in this article, or claim that may be made by its manufacturer, is not guaranteed or endorsed by the publisher.
